# Characterization and optimization of biogenic copper nanoparticles synthesized by *Pseudomonas putida* with cytocompatibility investigation

**DOI:** 10.1038/s41598-025-17705-8

**Published:** 2025-09-12

**Authors:** Osama El-Sayed, Basma T. Abd-Elhalim, Mohamed A. Mosa, Enas A. Hassan

**Affiliations:** 1 Department of Agriculture Microbiology, Faculty of Agriculture, Ain Shams University, Hadayek Shubra El-Khaimah, Cairo, 11241 Egypt; 2 Plant Pathology Research Institute, Agricultural Research Center, Giza, 12619 Egypt; 3 Nanotechnology and Advanced Nano-Materials Laboratory (NANML), Mycology and Disease Survey Research Department, Plant Pathology Research Institute, Agricultural Research Center, Giza, 12619 Egypt

**Keywords:** Copper nanoparticles (CuNPs), Cytotoxicity, Fourier transmission infrared (FTIR), Green synthesis, *Pseudomonas Putida*, Surface plasmon resonance (SPR), High-resolution transmission electron microscope (HR-TEM), Biotechnology, Microbiology, Nanoscience and technology

## Abstract

One area of science that is experiencing rapid growth is nanotechnology. The goal of nanotechnology research is to develop novel, economical, safe, and effective methods for creating nanoparticles. This study presents a green, cost-effective, and environmentally friendly method for synthesizing copper nanoparticles (CuNPs) using the bacterium *Pseudomonas putida*. The biosynthesis process was optimized by manipulating the growth medium with various carbon sources, resulting in Pp-CuNPs approximately 91.28 nm in diameter with a surface plasmon resonance (SPR) at 550 nm. Characterization techniques as Fourier transmission infrared (FTIR) and High-resolution transmission electron microscope (HR-TEM) confirmed the successful formation and stability of the nanoparticles, with a surface charge indicating good colloidal stability. Cytocompatibility assessments demonstrated high safety of Pp-CuNPs for lung Wi38 normal cell lines, supporting their potential applications in pharmaceutical, agricultural, and environmental fields. The findings underscore the feasibility and advantages of microbial-mediated copper nanoparticles (CuNPs) production, as well as their promising biomedical and ecological applications.

## Introduction

Nanotechnology has become a rapidly expanding field in recent years. Numerous potential applications in the fields of electronics, biology, chemistry, physics, antimicrobials, cosmetics, and agriculture have been described for nanoparticles (NPs)^1–4^. More emphasis has been paid to the advantages of metallic nanoparticles’ small size and large surface area for interactions^5–8^. Numerous chemical and physical methods were used to produce metal nanoparticles ^9^. However, the established chemical processes for producing nanoparticles can occasionally be prohibitively costly, time-consuming, hazardous, and chemically reliant. Therefore, methods for making nanoparticles that are quick, easy, affordable, and time-efficient are needed^10,11^.

Numerous metals, such as Ag, Cu, Fe, Au, and others, have been widely employed in the ecologically benign synthesis of NPs by a variety of microbial metabolites^12,13^. Compared to conventional chemical processes, environmentally friendly NPs production use less harmful chemicals^14,15^. Although the widespread use of chemically manufactured products in modern society has spurred technological advancement, concerns about their negative consequences have led to a search for natural alternatives. Microbial metabolites are a potential source of value-added molecules that can replace their synthetic counterparts. Microorganisms, including bacteria, fungi, and algae, can convert both typical and unconventional substrates into these beneficial chemicals^16,17^.

Although research into the nanoformulation of microbial metabolic extracts is still ongoing, the specific methods and uses may vary depending on the plant source and the desired outcome^18,19^. Microbial metabolite-mediated nano preparations can be designed to release active ingredients gradually and under control by modifying the size, content, or surface characteristics of the microbial-mediated nanoparticles, which will enable customized release kinetics.

Despite resource limitations, biosynthesis of metal nanoparticles promotes sustainable development since green nanomaterials made with microorganisms are thought to be safe, environmentally benign, and practical alternatives to costly physical or chemical processes^20,21^. Biogenic metal nanoparticles can be produced by bacteria using either extracellular or intracellular processes. Ions are broken down in the intracellular situation, whereas metal ions are broken down by substances in the extracellular situation^22–28^. Numerous studies investigate how the biomolecules and polysaccharides found in microbial extracts can function as reducing, capping, and chelating agents during the manufacture of nanoparticles with the goals of extending stability, preventing aggregation, and enhancing antibacterial and antifungal activity^29^.

According to^30–33^, the biosynthesis of CuNPs is carried out by bacteria such as *Agrobacterium* sp., *Bacillus megaterium*, *B. subtilis*, *Serratia* sp., *Pseudomonas stutzeri*, and *P. fluorescens*, with various carbon and nitrogen sources being important variables, as demonstrated by Araya-Castro et al.^34^.

The goal of the work was to use *P. putida* to produce copper nanoparticles (Pp-CuNPs) in an easy and environmentally friendly manner. The growth medium for *P. putida* could be changed to maximize the synthesis of Pp-CuNPs. The Pp-CuNPs have a high level of biosafety on lung Wi38 normal cell lines. The results obtained offer a high chance for a thorough investigation of Pp-CuNPs to be used in a variety of industries, including agriculture, medicine, and optics. The development of eco-friendly synthetic methods for CuNPs is crucial due to several limitations associated with conventional chemical synthesis techniques. Traditional chemical methods often involve the use of hazardous reducing agents, toxic solvents, and stabilizers, which pose environmental and health risks. These methods can be energy-intensive, expensive, and may generate toxic by-products, making them less sustainable and failing to meet the principles of green chemistry. In contrast, biological methods utilizing microorganisms such as bacteria, fungi, and algae offer a sustainable alternative. These biogenic approaches leverage natural metabolic processes to reduce metal ions into nanoparticles under mild conditions without the need for harmful chemicals. This eco-friendly strategy minimizes the ecological footprint, reduces chemical waste, and enhances safety for both the environment and human health. Furthermore, microbial synthesis ensures better control over nanoparticle size, shape, and stability due to the presence of biomolecules that act as reducing and capping agents. Such naturally synthesized CuNPs are biocompatible and suitable for biomedical, agricultural, and environmental applications, aligning with the growing demand for sustainable nanotechnology solutions. Therefore, advancing green biosynthesis methods is essential to develop safer, cost-effective, and environmentally friendly copper nanomaterials with broad potential uses.

The novelty of this work lies in its comprehensive approach to biosynthesizing copper nanoparticles (CuNPs) using *P. putida*, incorporating the optimization of culture conditions—such as specific nitrogen and carbon sources—that directly influence nanoparticle yield, stability, and size. Unlike prior studies that often focus solely on chemical synthesis or limited biological methods, this research combines environmentally friendly microbial biosynthesis with detailed physicochemical characterization and evaluation of biocompatibility. Notably, it provides new insights into the safe application of biologically synthesized CuNPs, demonstrating their stability, surface properties, and cytocompatibility with lung cell lines, which paves the way for biomedical and environmental uses. This integrated, sustainable, and biocompatible synthesis strategy, coupled with optimization techniques, represents a significant advancement and offers a novel platform for scalable green nanoparticle production with versatile applications.

## Materials and methods

### Chemicals used

All chemicals, such as copper sulfate (CuSO₄), salts, carbon sources (sucrose, lactose, fructose, galactose, glycerol, mannitol, and maltose), and nitrogen sources (peptone, beef extract, malt extract, tryptone, yeast, ammonium chloride, ammonium citrate, ammonium nitrate, and ammonium phosphate), are analytical grades that were purchased from Beta Lab, India.

### Media used

Medium (1): King’s B medium^35^ was used for the culture and preservation of *Pseudomonas* spp. Medium (2): Glucose copper modified (GCM) medium (agar and broth) was used for copper nanoparticles synthesis according to^36^.

### Source of the bacterial isolate

A bacterial isolate of *Pseudomonas* sp. was collected from the Plant Pathology Institute, Agriculture Research Center (ARC), Giza, Egypt. The bacterial isolate was periodically re-cultured and maintained using Med. (1) and stored at 4 °C.

### Standard inoculum

The standard inoculum of the *Pseudomonas* sp. was obtained by inoculating 50 ml of Med. (1) in an Erlenmeyer flask (250 ml) with a loopful of the culture and incubating at 30 °C at 150 rpm for 24 h. One milliliter of the inoculum contained 2.13 × 10^8^ CFU/ml.

### Evaluation of CuNPs biosynthesis by *Pseudomonas* sp. (Pp-CuNPs)

For the detection of Pp-CuNPs synthesis, the GCM medium (Med. 2) was prepared according to^37^, and after solidification, a fresh culture of bacterial isolate was streaked on the surface of the GCM medium. The inoculated plates were incubated at 30 °C for 24 h in an inverted position. The positive result was observed by the development of the brown shaded colonies on the surface of culture plates, which indicated the green synthesis of Pp-CuNPs, according to^38^. To confirm that the produced shaded brown color is not revealed from bacterial pigmentation behavior, the colonies were re-cultured onto Med. (1) plates (as a control). For the quantitative assay of Pp-CuNPs biosynthesis as described by^35^, flasks containing 50 ml of GCM broth medium were inoculated with 0.5 ml of standard inoculum of *Pseudomonas putida*, then placed on a shaking incubator (STIK Instrument 1050, Shanghai, China) at 150 rpm at 30 °C for 24 h. The synthesis of Pp-CuNPs was indicated by the color change of the medium from pale yellow or deep green to brown. Then the growth culture was centrifuged at 10,000 rpm for 10 min. Using a SIGMA 2–16 P centrifuge. The growth pellet of *Pseudomonas* sp. was discarded, whereas the supernatant was collected to investigate the physicochemical characteristics of Pp-CuNPs produced.

### Identification of *Pseudomonas* sp. isolate

According to^36^, a potent bacterial isolate was identified using the second edition of Bergey’s Manual of Systematic Bacteriology after conducting a thorough examination of its morphological and physiological characteristics. These findings were confirmed by the 16S rRNA sequencing in reference to^37^. The 16S rRNA gene sequence was analyzed using BLAST and a neighbor-joining method to identify the closest homologous bacterial members.

## Influence of medium components on Pp-CuNPs synthesis using individual factor at a time (IFAT) protocol

### Influence of the carbon source

Seven different carbon sources were used to investigate the carbon replacement effect on the Pp-CuNPs green synthesis using GCM medium (Med.2). Glucose, the essential carbon source in the synthesis medium, was replaced with a similar concentration of each of the tested carbon sources (sucrose, lactose, fructose, galactose, glycerol, mannitol, and maltose) to prepare carbon source modified (CSM) medium (Med.3). After that, the most suitable carbon source(s) was selected, and various concentrations of the most suitable carbon source, ranging between 0.5 and 2.5%, were tested using Med.2 with incubation at 30 °C for 24 h at 150 rpm.

### Influence of the nitrogen source

To study the impact of nitrogen sources in Pp-CuNPs biosynthesis, nine different nitrogen sources were replaced with the base nitrogen source in GCM medium (beef extract and peptone). The nitrogen sources were replaced with an equivalent concentration of each of the tested nitrogen sources [organic: peptone, beef extract, malt extract, tryptone, and yeast extract; inorganic: ammonium chloride, ammonium citrate, ammonium nitrate, and ammonium phosphate] with incubation at 30 °C for 24 h at 150 rpm. Following nitrogen source(s) selection, various concentrations of the most preferable nitrogen source, ranging between 0.1% and 1.25%, were tested.

### Characterization of Pp-CuNPs biosynthesis

UV–Vis spectral (Unico-UV 2100) was used to detect the development of Pp-CuNPs in the culture supernatant at a wavelength of λ 400–700 nm against deionized water as a blank^30^. According to^26^, using a dynamic light scattering (DLS) device (Malvern Zetasizer ZS90, UK, outfitted with BI-9000AT Digital Autocorrelation Version 2.0 software), the particle size and zeta potential for the suspension of CuNPs were ascertained. The obtained Pp-CuNPs solution was characterized using a transmission electron microscope (TECNAI 10-TEM, Philips, Amsterdam, The Netherlands) as follows: a drop of the solution was placed on the carbon-coated copper grids (CCG) and dried by allowing water to evaporate at room temperature^38^. Electron micrographs were obtained using a JEOL JEM-1010 transmission electron microscope at 80 kV at the Regional Center for Mycology and Biotechnology (RCMB), Al Azhar University. The Fourier-transform infrared spectroscopy (FTIR) measurement was carried out through the KBr pellet (FTIR grade), which was mixed with a pinch of potassium bromide (Himedia FTIR grade) in a crucible and was made into pellets by hydraulic press. The formed pellets were then analyzed using Fourier transform infrared (FTIR) spectrophotometry (Avatar-300, Nicolet, Green Bay, WI, USA). The chemical functional groups that induced the Pp-CuNPs to interact were identified at wavelengths between 400 and 4000 cm⁻¹ by KBr pellet.

### Cytocompatibility of Pp-CuNPs

The biosynthesized Pp-CuNPs cytotoxicity was evaluated with lung Wi38 normal cell lines cell viability using the MTT (3-(4,5-dimethylthiazol-2-yl)-2,5-diphenyl tetrazolium bromide) assay at Science Way Laboratories for Scientific Services, Cairo, Egypt, according to^39^. The suspension cells on the 96-well plate were spun at 2500 rpm for 5 min at 4 °C in a microplate-compatible centrifuge (CAPPRondo Microplate Centrifuge, Germany) and properly aspirated in order to calibrate before the MTT experiment. Make sure that each sample has the same amount of serum-free medium. Next, fill each well with 50 µl of serum-free medium and 50 µl of MTT solution. For three hours, incubate the plate at 37 °C. The lung Wi38 normal cell lines were consistently growing in Roswell Park Memorial Institute (RPMI) medium. Penicillin G sodium (100 units/ml), 2 mM l-glutamine, 10% fetal bovine serum (FBS), and 100%. After sub-confluence, cells were maintained at 37 °C in humidified air containing 5% CO₂. Following treatment with trypsin and EDTA at 37 °C, monolayer cells were gathered for subculturing. Cells were employed when confluence reached 75%. To treat the cells, another aliquot of 100 µl of the medium with various medication dosages was utilized. The medium was thrown out after the drug exposure of 48 h, and 20 µl of the 1 mg/ml stock solution was mixed with 100 µl of phosphate buffer solution (PBS) in each well. Then this mixture was incubated for 4 h at 37 °C. After that, 100 µl of pure dimethyl sulfoxide (DMSO) was used to dissolve the formazan crystals that had formed. The formazan solutions’ absorbance was determined at λmax 570 nm by the utilization of an ELISA plate reader (FLUOstar OPTIMA, BMG LABTECH).

### Statistical analysis

The data obtained were statistically analyzed using OriginPro 2024 software and SPSS V.19.0, and the average and standard deviation were calculated. The IC_50_ dosage was calculated using Pad Prism software. All experiments are conducted in *n* = 3 runs.

## Results

### Identification of *Pseudomonas* sp. isolate

**Fig. 1:**
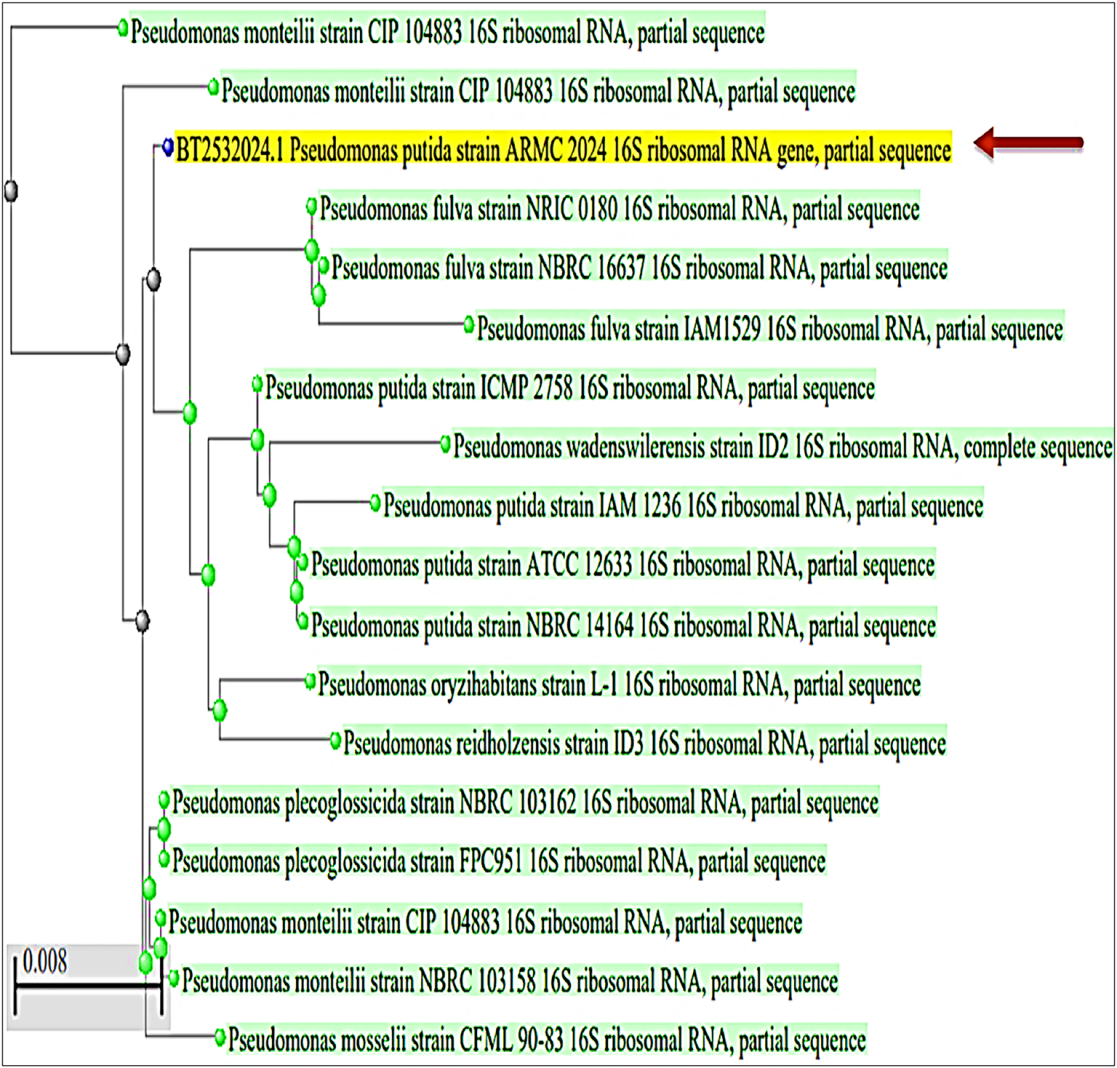
Phylogenetic tree of *Pseudomonas* sp. isolate showing 99% similarity to *P. putida*.

The* Pseudomonas* sp. isolate was identified based on its culture growth pattern, cell morphology, and biochemical properties, based on its phenotypic characteristics. Colonies of *Pseudomonas* sp. isolate grown on Med. (1) are round, with a smooth surface and edges, with soluble yellow, fluorescent pigment. *Pseudomonas* sp. isolate, a Gram-negative, small, short, rod-shaped, non-spore-forming bacterium, exhibits aerobic properties and positive results for casein, starch, and lipid hydrolysis. The 16S rRNA gene sequence analysis of *the Pseudomonas* sp. isolate reveals it belongs to the genus *Pseudomonas* and is closely related to *Pseudomonas putida* (99% similarity), as shown in Fig. 1.

### Detection of CuNPs biosynthesis using *P. Putida* (Pp-CuNPs)

**Fig. 2:**
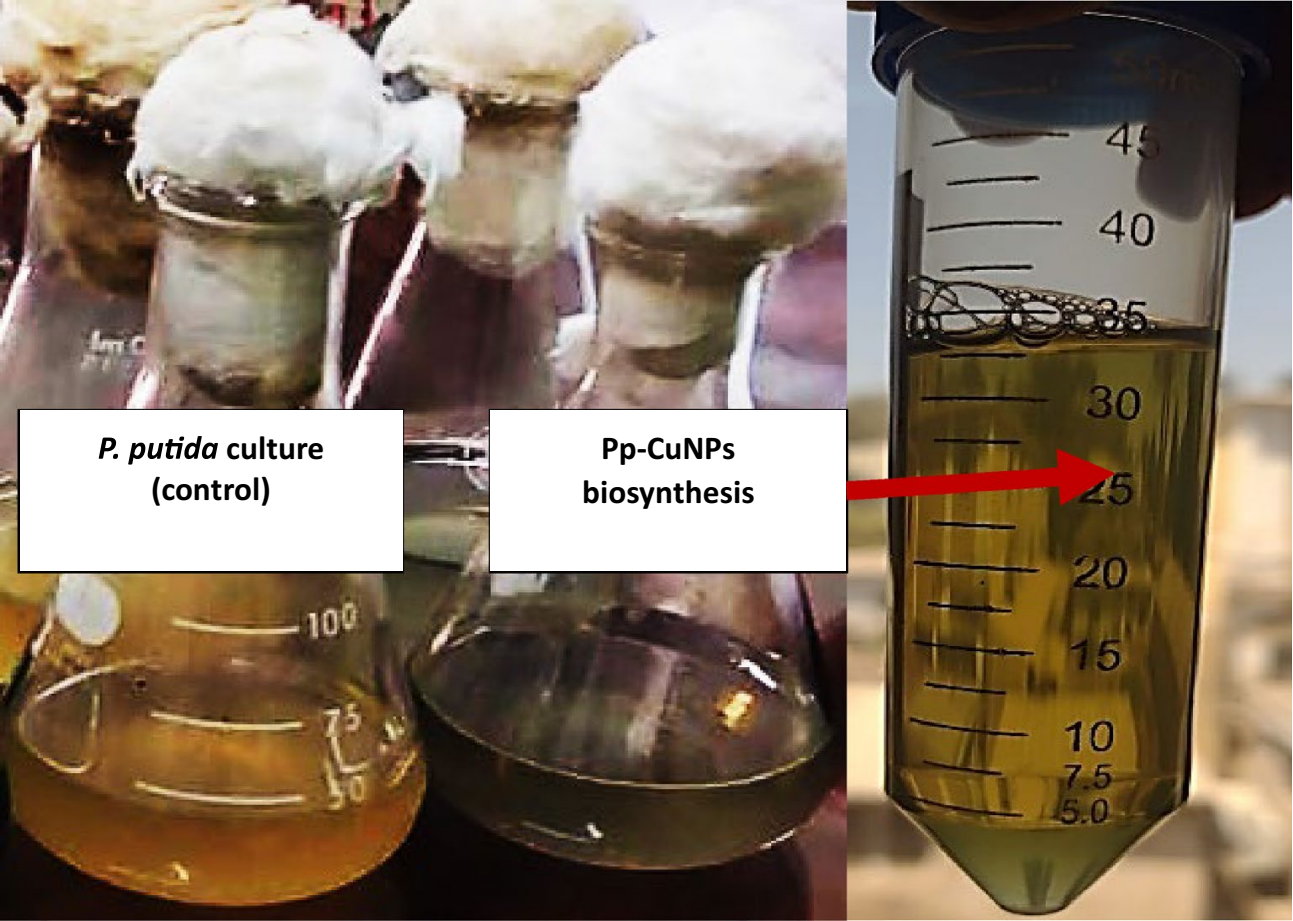
Indication of Pp-CuNPs formation by *P. putida* cultivated on GCM broth medium incubated at 30 °C for 24 h.

The primary indication of Pp-CuNPs biosynthesis was the brown zone formation surrounding the *P. putida* growth colony grown on GCM agar medium (Med. 2) incubated at 30 °C for 24 h. The color change of the *P. putida* growth culture from yellow to greenish due to the SPR excitation confirms Pp-CuNPs biosynthesis (Fig. 2).

### Quantitative evaluation of Pp-CuNPs biosynthesis

**Fig. 3:**
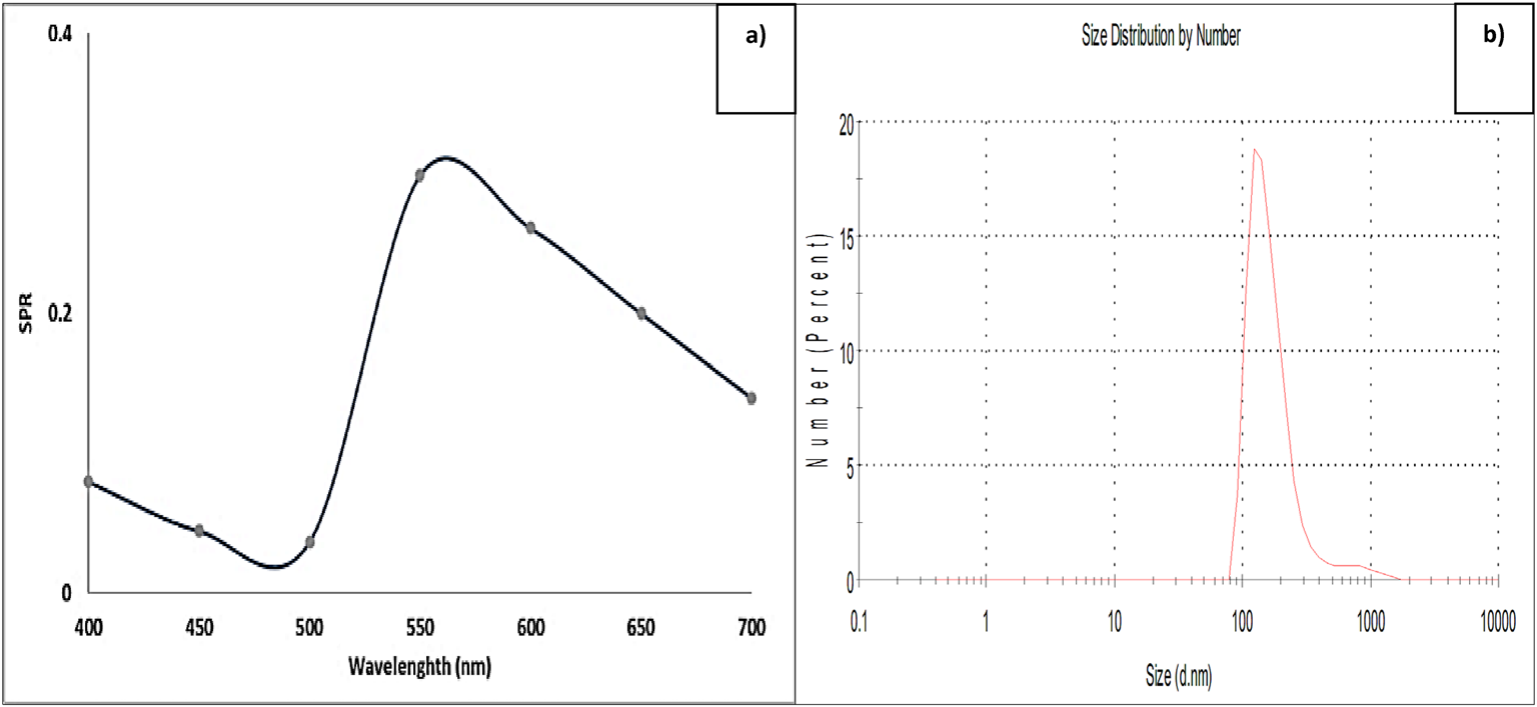
**a)** UV–visible spectrum profile, **b)** DLS profile of Pp-CuNPs formed by *P. putida* incubated at 30 °C for 24 h, cultivated on GCM broth medium.

The obtained supernatant from the *P. putida* culture was examined using the UV–Vis spectrophotometer at wavelengths from 400 to 700 nm. The results, according to Fig. 3a, showed an SPR score of 0.30 at wavelength 550 nm, the associated wavelength for Pp-CuNPs formation. In addition, the Pp-CuNPs formation was confirmed using DLS characterization to measure the particle size for the biosynthesized Pp-CuNPs. As shown in Fig. 3b, the Pp-CuNPs size is 102.3 nm.

## Influence of medium components on Pp-CuNPs synthesis using individual factor at a time (IFAT) protocol

### Influence of the carbon source

**Fig. 4:**
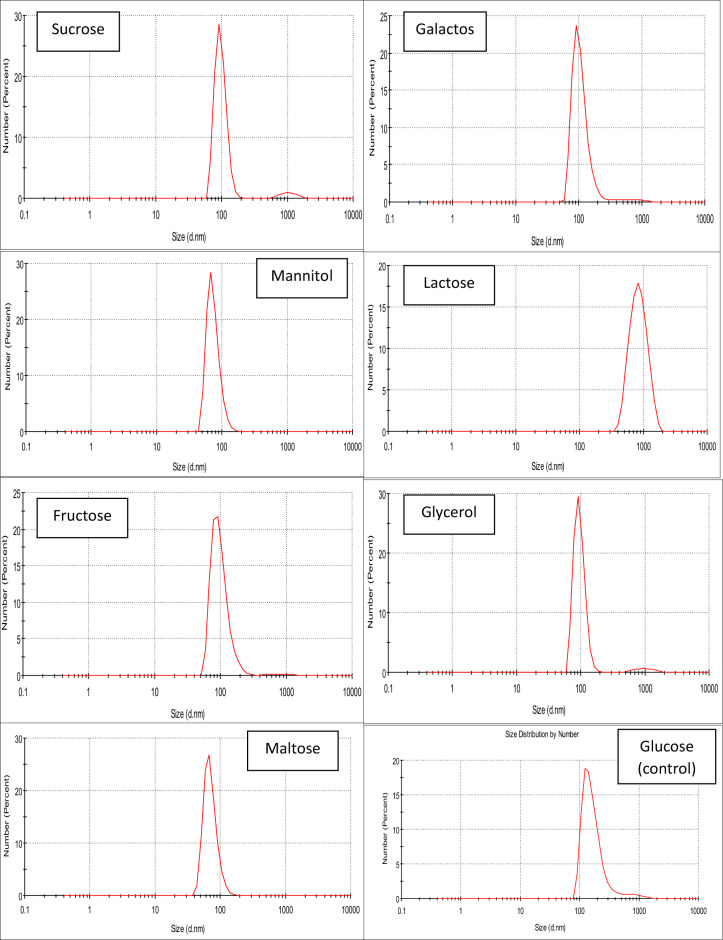
Carbon sources screening of Pp-CuNPs formation using *P. putida* incubated at 30 °C for 24 h, cultivated on glucose copper modified (GCM) medium.

As mentioned in Fig. 4, the order of Pp-CuNPs size diameters using different carbon sources was arranged ascendingly as follows: mannitol & maltose (68.06 nm), fructose, galactose, glycerol & sucrose (91.28 nm), and lactose (825.0 nm) compared to glucose as a control (102.3 nm).

### The most effective carbon source(s) concentrations for Pp-CuNPs biosynthesis

**Fig. 5:**
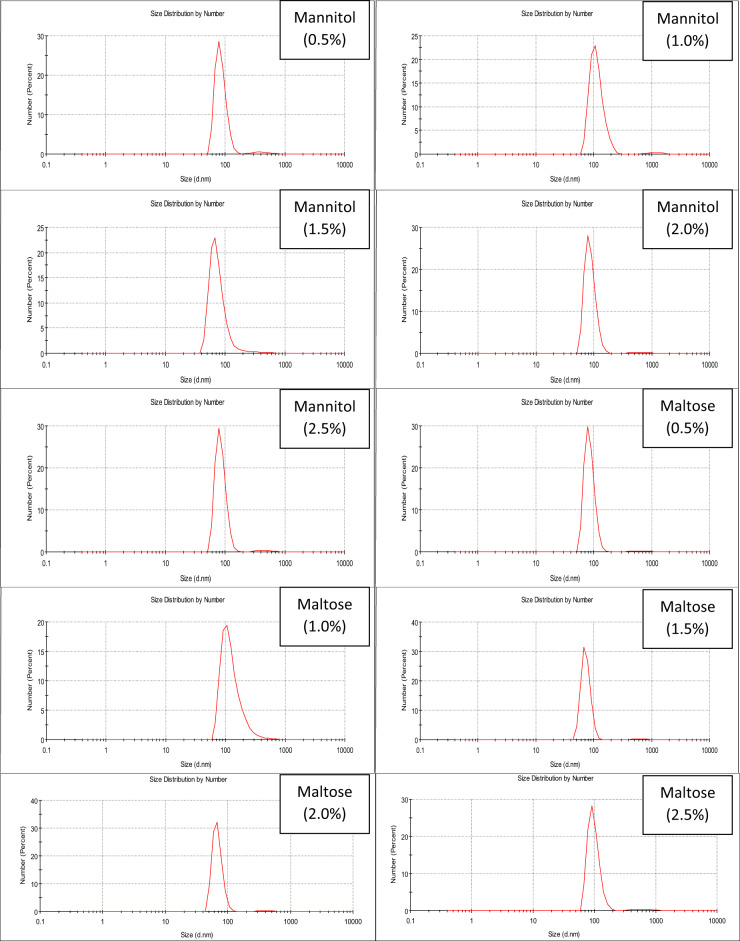
DLS screening of Pp-CuNPs formation by *P. putida* using the different concentrations of the most potent carbon source, incubated at 30 °C for 24 h, cultivated on glucose copper modified (GCM) medium.

As mentioned in Fig. 5, the impact of effective carbon source (mannitol and maltose) concentrations between 0.5% and 2.5% on Pp-CuNPs biosynthesis was as follows: 78.82 nm, 68.06 nm, 68.06 nm, 78.82 nm, and 78.82 nm with mannitol & 78.82 nm, 68.06 nm, 68.06 nm, 68.1 nm, and 91.28 nm for maltose at 0.5, 1.0, 1.5, 2.0, and 2.5%, respectively. From the previous results, we could conclude that the most suitable carbon source concentration was 1% for mannitol or maltose, as it recorded the lowest particle size, and the concentrations more or less than this concentration led to an increase in Pp-CuNPs diameter.

### Influence of the nitrogen source

**Fig. 6:**
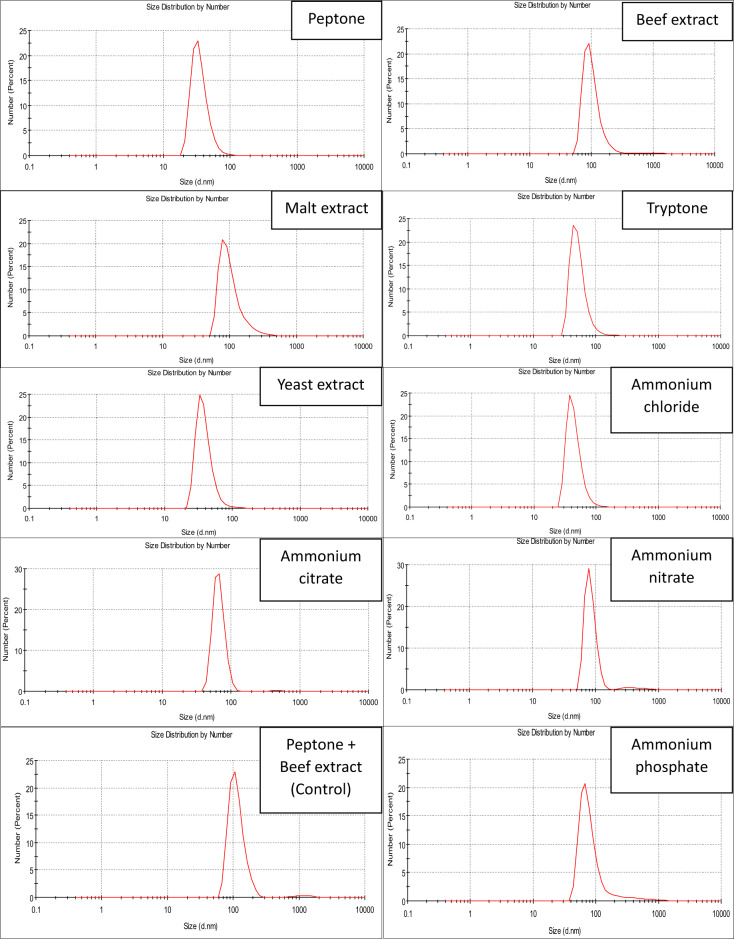
DLS screening of Pp-CuNPs formation using the different nitrogen sources using *P. putida* incubated at 37 °C for 24 h using glucose copper modified (GCM) medium.

After selecting the appropriate carbon source (1% mannitol), the effect of nitrogen sources on the biosynthesis of Pp-CuNPs was investigated. As mentioned in Fig. 6, the order of size diameters using various nitrogen sources was as follows: 32.67 nm with yeast extract or peptone & 43.82 nm with malt extract, but tryptone and ammonium sulfate recorded 78.82 nm. Pp-CuNPs with ammonium chloride have the highest diameters (91.45 nm), and ammonium nitrate & ammonium phosphate and the control sample recorded 68.06 nm.

### The most effective nitrogen source(s) concentrations for Pp-CuNPs biosynthesis

**Fig. 7:**
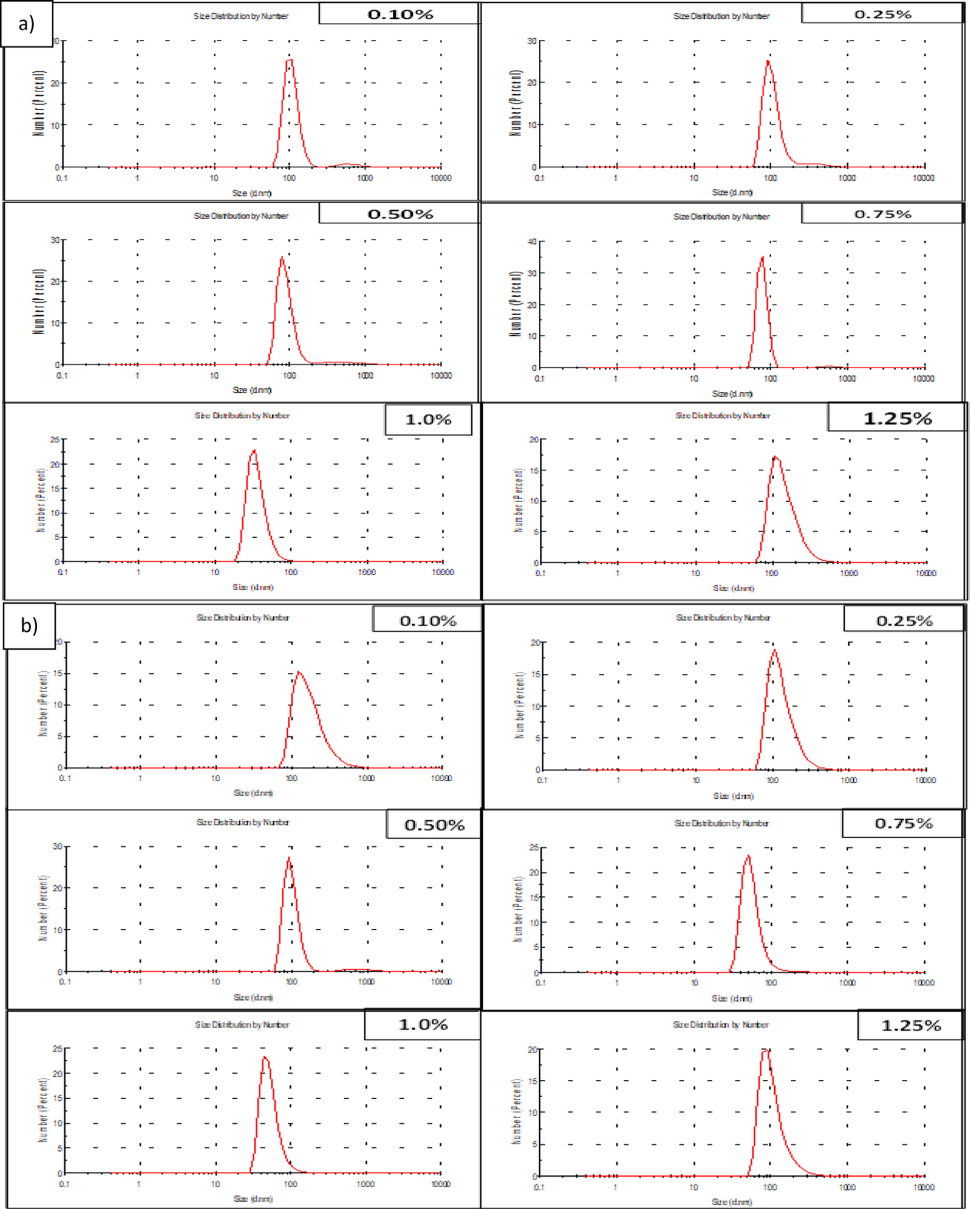
DLS screening of Pp-CuNPs formation using the different nitrogen source concentrations using *P. putida* incubated at 30 °C for 24 h, cultivated on glucose copper modified (GCM) medium. **a)** Peptone, **b)** Yeast extract.

Following nitrogen source(s) selection, various concentrations of the most effective nitrogen source (yeast extract & peptone), ranging between 0.1% and 1.25%, were tested using the GCM broth medium (Fig. 7). The yeast extract concentrations revealed diameters of 78.82 nm (0.1%), 78.82 nm (0.25%), 105.7 nm (0.50%), 91.28 nm (0.75%), 32.67 nm (1.0%), and 105.7 nm (1.25%). For peptone, the various concentrations gave size diameters of 91.28 nm (0.1%), 50.75 nm (0.25%), 122.4 nm (0.50%), 105.7 nm (0.75%), 32.67 nm (1.0%), and 91.28 nm (1.25%). From the obtained results, we could conclude that the most suitable nitrogen source concentration was 1% peptone, as it could maintain the diameter, as observed with the suitable carbon source (mannitol), and at concentrations lower or higher than the suitable concentration, the Pp-CuNPs diameter will increase. So, after the IFAT optimization for carbon and nitrogen sources, we can conclude that 1% mannitol and 1% peptone are the most nutritional factors that affect Pp-CuNPs biosynthesis by *P. putida*.

### Characterization of the biosynthesized Pp-CuNPs

#### HR-TEM investigation

**Fig. 8:**
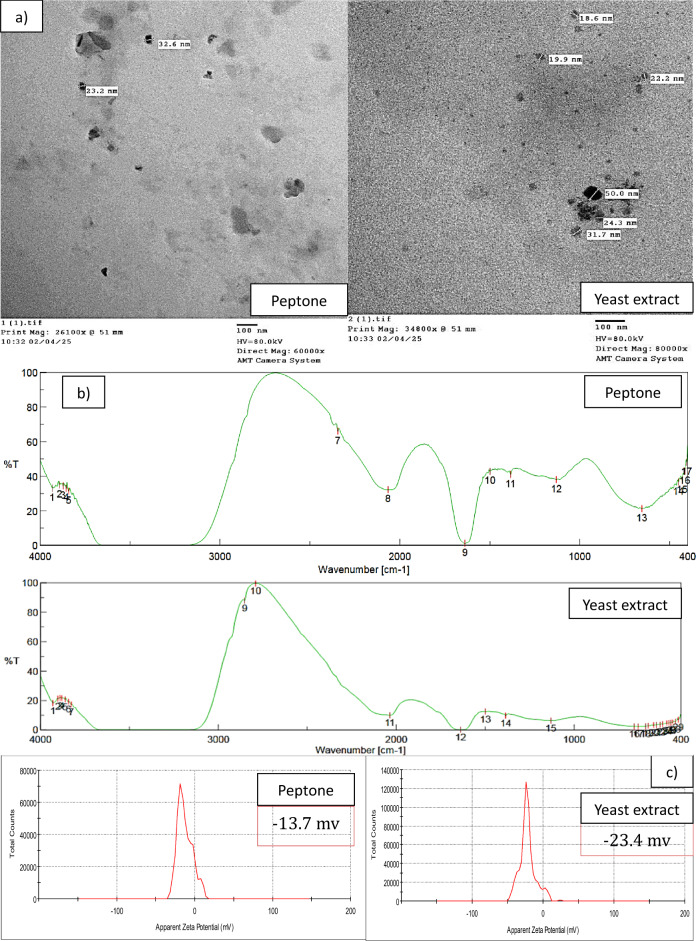
Different characterizations of Pp-CuNPs: **a)** HR-TEM, **b)** FTIR spectra, and **c)** Zeta potential.

HR-TEM revealed that the biosynthesized Pp-CuNPs had a semi-spherical shape with size ranges between approximately 23.2 nm and 43.9 nm when synthesized using yeast extract as the carbon source and between 18.6 nm and 25.9 nm with peptone (Fig. 8a). The nanoparticles exhibited a coat-shell structure, indicative of protein capping around the particles, which contributes to their stability. These morphological features confirm the successful biosynthesis of uniform and nanoscale copper nanoparticles.

### FTIR investigation

The FTIR examination confirmed O-H stretching and amine binding groups for both peptone and yeast extract, indicating the protein coverage and high negative charge corresponding to the stability. As shown in Fig. 8b, with peptone, characteristic peaks of O–H stretching of alcohol (3432.67 cm⁻¹), O=C=O stretching of carbon dioxide (2345.02 cm⁻¹), N=C=S stretching of isothiocyanate (2066.35 cm⁻¹), N–H bending of amine (1638.23 cm⁻¹), C–H bending of alkane (1499.38 cm⁻¹), O–H bending of phenol (1384.64 cm⁻¹), C–N stretching of amine (1130.08 cm⁻¹), and C–Cl stretching of halo compound (655.679 cm⁻¹) were obtained. In the case of yeast extract, more associations were obtained for O–H stretching of alcohol (3285.14 cm⁻¹), O–H stretching of carboxylic acid (2852.2 cm⁻¹), C–H stretching of aldehyde (2790.49 cm⁻¹), N=C=S stretching of isothiocyanate (2036.46 cm⁻¹), N–H bending of amine (1638.23 cm⁻¹), C–H bending of alkane (1499.38 cm⁻¹), O–H bending of phenol (1384.64 cm⁻¹), C–N stretching of amine (1131.05 cm⁻¹), C–Cl stretching of halo compound (661.464 cm⁻¹), and C–I stretching of halo compound (640.251 cm⁻¹).

### Zeta potential

As shown in Fig. 8c, the Pp-CuNPs zeta potential investigation scored − 13.7 and − 23.4 mV for peptone and yeast extract, respectively.

### Cytocompatibility of the biosynthesized Pp-CuNPs

**Fig. 9:**
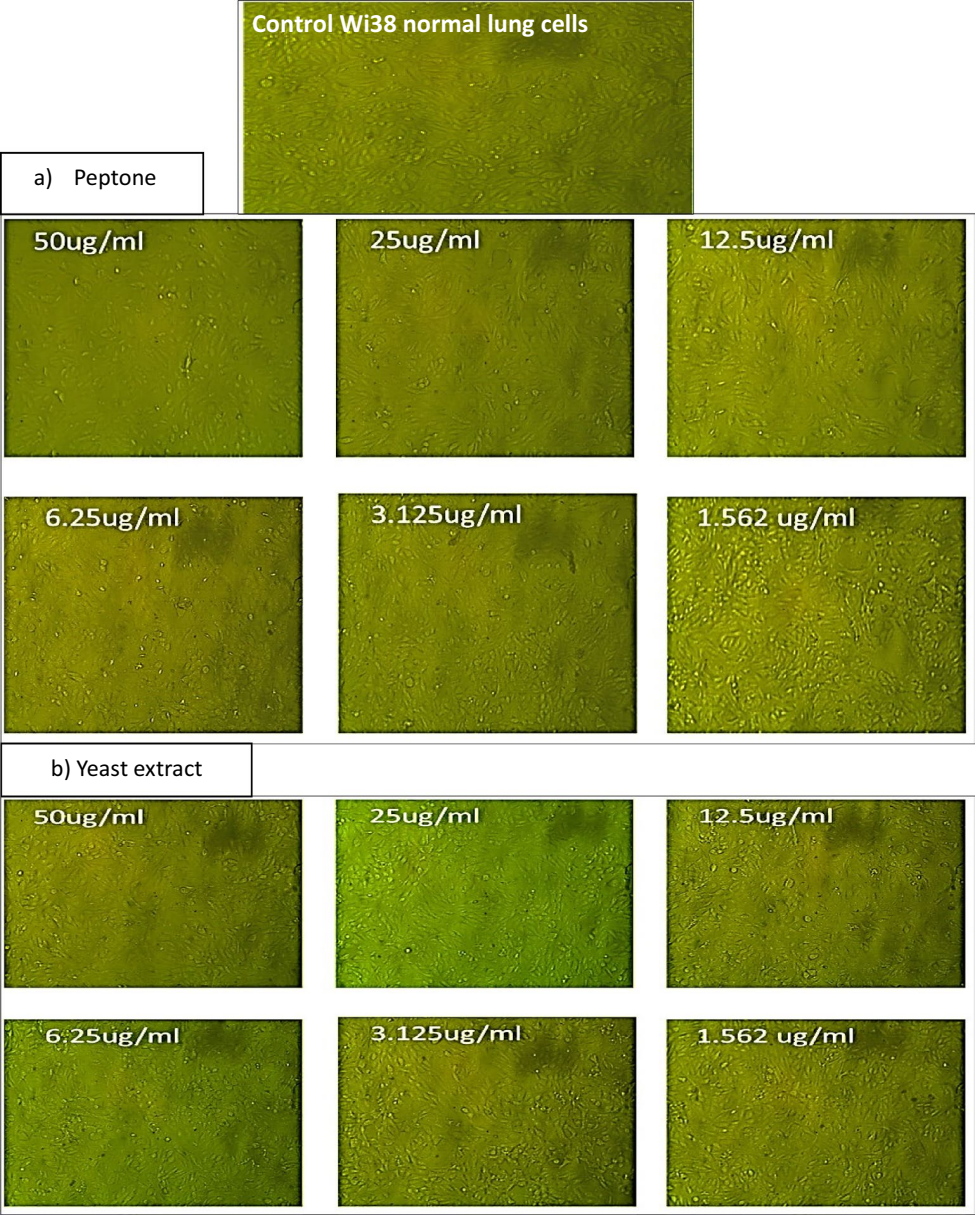
Cytocompatibility of the biosynthesized Pp-CuNPs **a**) Produced in the presence of peptone and **b**) Produced in the presence of yeast extract (as a nitrogen source) on normal lung Wi38 cell lines at different concentrations.

The cytocompatibility activity assay using lung Wi38 normal cell lines revealed that Pp-CuNPs were safe in high concentrations up to 50 µg/ml, as shown in Fig. 9a and b. The cell morphology of normal lung cell lines over the different concentrations didn’t reveal any cell changes, indicating high biocompatibility.

## Discussion

In materials science, green synthesis of metal nanoparticles is a dependable, sustainable, and eco-friendly method. Active metabolites used in copper nanoparticles produced by bacteria, fungi, algae, and plants serve as reducing and stabilizing agents^30,32,40^. The study verified that *P. putida* biosynthesized CuNPs by seeing a color shift from yellow to greenish on the broth medium and UV–Vis spectrophotometer results at the SPR of 0.30 at wavelengths of 550 nm. According to a previous study, a color change seen in a flask containing *Morganella* sp. and *Stereum hirsutum* indicates that microbial metabolisms are the main source of CuNPs from CuSO₄ solution^24^. The cell-free supernatant of *P. silesiensis* was also reported to be used to create CuNPs, which changed color from blue to dark green and had a distinct absorption peak in the UV–Vis absorption spectrum^23^. The produced Pp-CuNPs displayed UV–visible absorption maxima close to 540 and 630 nm, as reported in a previous study^40^. According to several studies, biological molecules are in charge of the production of CuNPs, which is accomplished by enzymatic mechanisms involving reductases and aggregation with other cellular proteins for metal ion reduction and NPs stability^41,42^. An enzymatic mechanism involving reductases reduces metal ions extracellularly and stabilizes the NPs. This is followed by aggregation with other cellular proteins as investigated in *Pseudomonas* sp. growth cultures^33,43,44^.

To determine the relationship between bacterial growth and medium components during *P. putida* production of CuNPs, several carbon and nitrogen sources were examined. For galactose, mannitol, fructose, glycerol, and maltose, the size diameters utilizing various carbon sources revealed size reduction with percentages of 10.8, 33.5, 10.8, 10.8, and 33.5%, respectively. To assess each carbon source’s efficiency, the percentage decrease was calculated. In contrast, the diameter size increased by 8.04 times using lactose . Mannitol or maltose was shown to be an efficient carbon source that affected the production of Pp-CuNPs. At 1%, mannitol was determined to be the best carbon source. The effect of nitrogen sources on Pp-CuNPs biosynthesis showed that the best nitrogen sources were peptone and yeast extract, as seen by a 52% decrease in diameter reduction as compared to the control sample. With diameters comparable to mannitol, 1% peptone was the most efficient nitrogen source.

Proteose peptone, yeast extract, peptone, NaNO₃, and ZnNO₃ were among the nitrogen sources used in the synthesis of CuNPs, which were carried out with *P. fluorescens*, *Thermoanaerobacter* sp. X513, *B. subtilis* T-1, and *Chaetomium globosum*, respectively, according to^33,45–47^. In HR-TEM examination, Pp-CuNPs using peptone were found to be 23.2–43.9 nm, whereas yeast extract was found to be 24.3–50.0 nm. Both had a shell-covering layer on them and had a semi-spherical shape. CuNPs particles were earlier shown by Abd-Elhalim et al.^33^ as monodispersed spherical nanoparticles with an extraordinary, unified shape. Additionally, it was reported that *P. fluorescens* generated spherical CuNPs^43^. CuNPs, which are made with enzymes from biological cell-free extract, have spherical and hexagonal forms with an average particle size of 49 nm, according to Harne et al.^48^.

The FTIR analysis’s obtained spectra reveal the molecular connections between CuNPs and the constituents of the fermentation medium. Protein coverage and stability were shown by the FTIR analysis, which verified O-H stretching and amine binding groups in both peptone and yeast extract. There were distinct peaks in the yeast extract for alcohol, carboxylic acid, aldehyde, isothiocyanate, amine, alkane, phenol, halo compound, and C-I stretching, as well as in the peptone for alcohol, carbon dioxide, isothiocyanate, amine, alkane, phenol, and halo compound. Different amide and amine peaks were discovered in CuNPs by Abd-Elhalim et al.^33^, indicating the existence of proteins. According to Jang et al.^46^, proteins that envelop CuNPs enhance their long-term stability and decrease oxidation.

Strong stability during reduction was shown by the zeta potential measurement, which showed that the peptone and yeast extract had charges of − 13.7 and − 23.4 mV, respectively. CuNPs had a zeta potential of − 19.6 mV, according to Tiwari et al.^40^, and an FTIR spectrum showed protein. Through electrostatic stabilization, the molecules’ non-ionic composition promotes long-term stability by preventing NP aggregation^33^.

According to Tiwari et al.^40^, Pp-CuNPs demonstrated safety against lung Wi38 normal cell lines at high dosages and preserved cell morphology at high doses up to 50 µg/ml, demonstrating that CuNPs are biocompatible. With an IC_50_ value of 1057.0 µg/ml, *P. silesiensis* CuNPs had a considerable effect on Wi38 cell viability, according to Abd-Elhalim et al.^33^. Biosynthesized nanoparticles that are mediated, coated, and stabilized by bioactive groups utilizing bacterial cell-free extracts are safer and more biocompatible, as proven by^49–51^. The biological and biocompatibility characteristics of biosynthesized nanoparticles are greatly influenced by their surface charge; negatively charged cellular membranes affect interaction, whereas positively charged nanoparticles are more harmful^52^.

## Conclusions

**Fig. 10:**
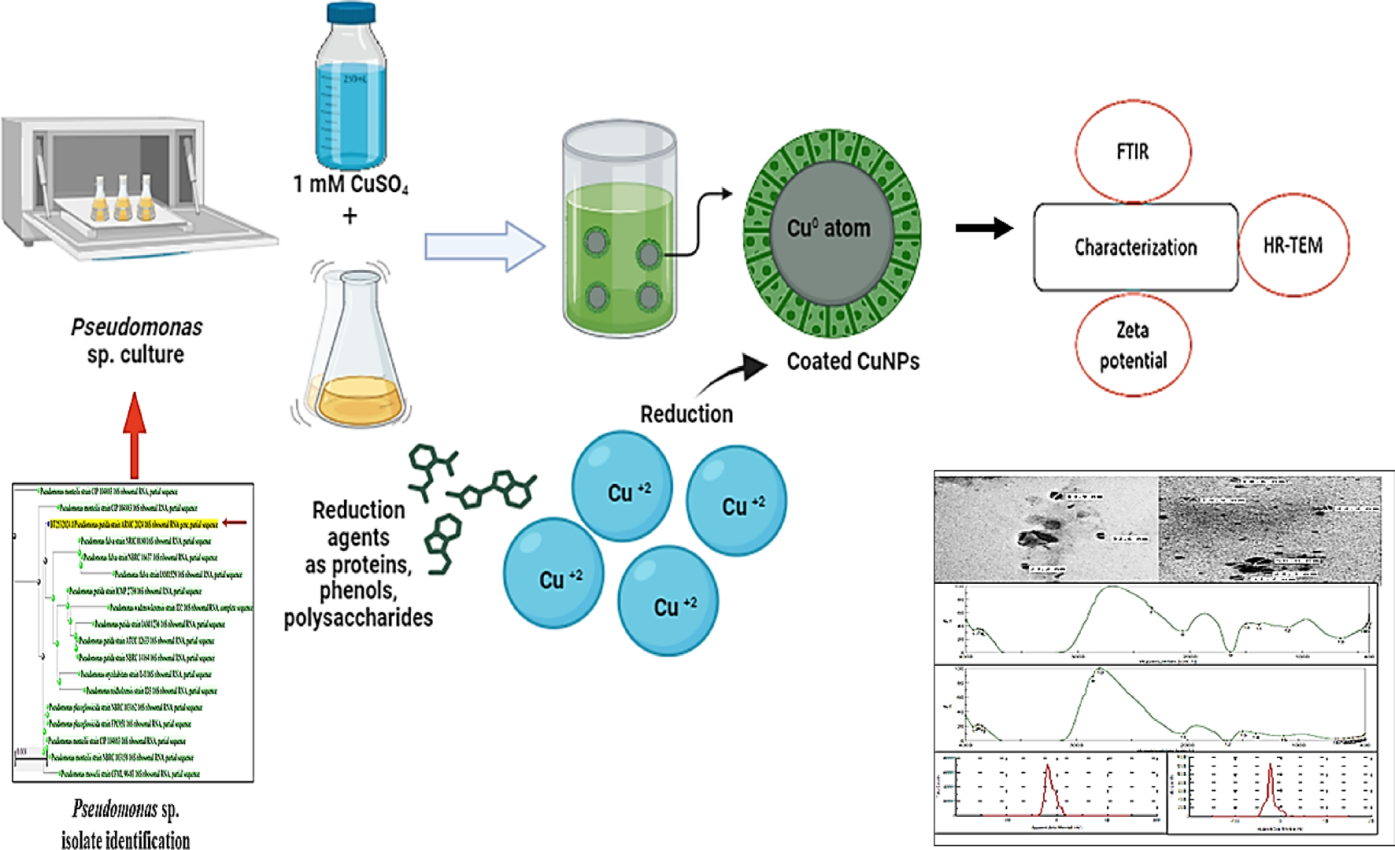
Graphical abstract of the Pp-CuNPs biosynthesis mechanism.

This study highlights the successful biosynthesis of copper nanoparticles (CuNPs) using *P. putida* (Fig. 10), emphasizing the importance of optimizing culture conditions—particularly nitrogen and carbon sources—to achieve stable, appropriately sized, and biocompatible nanoparticles. The characterized CuNPs demonstrated excellent stability, surface properties, and low cytotoxicity toward lung cell lines, underscoring their safety for potential biomedical applications. Moreover, the environmentally friendly biosynthesis approach offers significant advantages over traditional chemical methods, including cost-effectiveness and reduced ecological impact. These findings support the potential of *P. putida*-mediated CuNPs production for diverse applications across medicine, agriculture, and environmental remediation. Future work should focus on scaling up the process, thoroughly assessing long-term stability, and evaluating ecological effects to facilitate commercial development of these biogenic nanomaterials.

The study discusses several limitations and challenges associated with the biosynthesis and application of copper nanoparticles (CuNPs) using *P. putida*, such as: (1) stability of nanoparticles. While the zeta potential measurements indicated good stability, the long-term stability of the synthesized nanoparticles in various environments remains a concern. Factors such as pH, ionic strength, and the presence of other substances can affect their stability and functionality. (2) Scalability of production, as the biosynthesis process may face challenges when scaling up from laboratory conditions to industrial production. Ensuring consistent quality and yield of nanoparticles at a larger scale can be difficult. (3) Cytotoxicity and biocompatibility. Although the study investigates cytocompatibility, the potential cytotoxic effects of CuNPs on different cell types and their long-term effects in biological systems need further exploration. Understanding the interactions at the cellular level is crucial for safe applications. (4) Environmental Impact, the environmental implications of using metal nanoparticles, including their potential toxicity to non-target organisms and ecosystems, require thorough assessment. The biosynthesis process must be evaluated for sustainability and ecological safety. (5) Characterization techniques, while various characterization techniques were employed, the complexity of nanoparticle behavior in biological systems may not be fully captured by standard methods. Advanced characterization techniques may be necessary to understand the nanoparticles’ interactions in vivo.

## Data Availability

“*Pseudomonas putida* ARMC 2024 was deposited in the gene bank https://www.ncbi.nlm.nih.gov/nuccore/PP534171.”
